# Avian Influenza and UV-B Blocked by Biomass Smoke

**DOI:** 10.1289/ehp.113-a806b

**Published:** 2005-12

**Authors:** Forrest M. Mims

**Affiliations:** Geronimo Creek Observatory Seguin, Texas, E-mail: forrest.mims@ieee.org

Washam (2005) described various poultry inoculation strategies being considered for controlling the spread of avian influenza in Southeast Asia and China. Longini et al. (2005) proposed that a future avian influenza A pandemic might be contained at the source by targeted prophylaxis, quarantine, and prevaccination.

Washam (2005) correctly noted that “Asian farmers, though, are running out of options.” I propose a new option: Avian influenza might be controlled by a substantial reduction in regional scale biomass smoke in Southeast Asia that will allow natural solar ultraviolet-B radiation (UV-B) to suppress the virus before infection occurs.

Influenza viruses and various non-pigmented bacteria are killed by UV-B wavelengths in sunlight (Hollaender and Oliphant 1944). Biomass smoke significantly suppresses natural levels of UV-B, and severe smoke pollution reduced UV-B by up to 95% during the burning seasons in Brazil in 1995 (Mims 1996) and 1997 (Mims FM III, White B, unpublished data). Reduced UV-B on 6 days in August 1997 was well correlated (*r*^2^ = 0.83) with an increase in the ratio of nonpigmented bacteria vulnerable to UV-B to pigmented bacteria that are protected from UV-B (Mims and White 1998). Although airborne influenza viruses were not measured, 1997 hospital admission records at Alta Floresta, Brazil, showed that influenza incidence was highest during the burning season (de Castro GC, personal communication).

Human cases of avian influenza in Thailand and Vietnam peaked during the winter burning seasons of 2003 and 2004 (Thailand Ministry of Public Health 2005). Assuming similar optical properties of biomass smoke in Southeast Asia and Brazil, where UV-B and optical depth are highly correlated, optical depth measurements over Thailand and Vietnam by NASA’s Terra and Aqua satellites suggest highly suppressed UV-B during these avian influenza outbreaks (Mims FM III, unpublished data).

Human cases of avian influenza in Thailand and Vietnam since December 2003 have peaked during both the rainy season and the burning season. Thus, periods of prolonged cloudiness and severe smoke pollution could play a role in initiating avian and other influenza outbreaks by attenuating the solar UV-B that might otherwise suppress influenza viruses in outdoor air exposed to sunlight. The transmission of avian influenza to people during these periods is enhanced by the fact that poultry raised for human consumption are often kept within several meters of where people live (World Health Organization 2004).
